# Selection on milk production and conformation traits during the last two decades in Japan

**DOI:** 10.5713/ajas.18.0259

**Published:** 2018-07-26

**Authors:** Kenji Togashi, Takefumi Osawa, Kazunori Adachi, Kazuhito Kurogi, Kota Tokunaka, Takanori Yasumori, Tsutomu Takahashi, Kimihiro Moribe

**Affiliations:** 1 Maebashi Institute of Animal Science, Livestock Improvement Association of Japan, Maebashi, Gunma 371-0121, Japan; 2 National Livestock Breeding Institute, Nishigo-mura, Nishishirakawa-gun, Fukushima 961-8511, Japan; 3 Livestock Improvement Association of Japan, Koto-ku, Tokyo 135-0041, Japan

**Keywords:** Selection Differentials, Actual Yearly Genetic Gain, Milk Production Traits, Conformation Traits

## Abstract

**Objective:**

The purpose of this study was to compare intended and actual yearly genetic gains for milk production and conformation traits and to investigate the simple selection criterion practiced among milk production and conformation traits during the last two decades in Japan. Learning how to utilize the information on intended and actual genetic gains during the last two decades into the genomic era is vital.

**Methods:**

Genetic superiority for each trait for four paths of selection (sires to breed bulls [SB], sires to breed cows [SC], dams to breed bulls [DB], and dams to breed cows [DC]) was estimated. Actual practiced simple selection criteria were investigated among milk production and conformation traits and relative emphasis on milk production and conformation traits was compared.

**Results:**

Selection differentials in milk production traits were greater than those of conformation traits in all four paths of selection. Realized yearly genetic gain was less than that intended for milk production traits. Actual annual genetic gain for conformation traits was equivalent to or greater than intended. Retrospective selection weights of milk production and conformation traits were 0.73:0.27 and 0.56:0.44 for intended and realized genetic gains, respectively.

**Conclusion:**

Selection was aimed more toward increasing genetic gain in milk production than toward conformation traits over the past two decades in Japan. In contrast, actual annual genetic gain for conformation traits was equivalent to or greater than intended. Balanced selection between milk production and conformation traits tended to be favored during actual selection. Each of four paths of selection (SB, SC, DB, and DC) has played an individual and important role. With shortening generation interval in the genomic era, a young sire arises before the completion of sire’s daughters’ milk production records. How to integrate these four paths of selection in the genomic era is vital.

## INTRODUCTION

Many studies have examined genetic trends by regression of estimated breeding values on time [1–3]. Because gain in those studies was considerably less than what is possible under ideal circumstances, comparing actual selection practices and realized genetic gains with those intended during the past two decades is important to utilize the information in the past into the new genomic era. Firstly, to this end, we compared intended and actual yearly genetic gains for milk production (milk fat and protein yields) and conformation traits (overall feet and legs score, overall teat score, and classification final score) during the last two decades in Japan. Next, since there are four paths of selection for genetic improvement, such as sires to breed bulls (SB), sires to breed cows (SC), dams to breed bulls (DB), and dams to breed cows (DC) [4], we compared previously intended and actually practiced selection differentials for milk production and conformation traits for four paths of selection during the last two decades in Japan. Although selection index in retrospect is based on the assumptions that selection has been on an index, that the index used was the optimum one, and that all characters on which selection was based are included in the data set [5], we investigated selection index in retrospect [6,7] as a practical and simple selection criterion among milk production and conformation traits during the last two decades in Japan. Based on the index in retrospect, we compared the relative emphasis of selection regarding milk production and conformation traits between intended and realized genetic gains. The purpose of this study was to compare intended and actual yearly genetic gains for milk production and conformation traits during the last two decades in Japan, to compare the retrospective selection emphasis on those traits, and to provide insight into the new genomic era.

## MATERIALS AND METHODS

### Previously intended and actual realized genetic gains

Predicted genetic values were those calculated by using an animal model comprising 11,627 bulls and 4,051,752 cows [8]. Because milk fat yield (MF) and milk protein yield (MP), overall feet and legs score (FL), overall teat score (TE), and classification final score (FS) are major traits used as selection criteria in Japan [8], we considered MF and MP as traits representing milk production and FL, TE, and FS as conformation traits. The FS was derived as a total score from (frame×25)+ (feet and legs×20)+(dairy strength×15)+(udder×40), where frame, feet and legs, dairy strength, and udder ranged from 0.5 to 1.0. The selection index was composed of these five traits. Data were extracted for animals with differences in estimated breeding value (EBV) between the selected and cows born in the same year along four paths of selection (SB, SC, DB, and DC) and generation intervals, thus resulting in data for 1993 through 2012. Average EBVs of cows born in the same year as sires used or dams used were adopted as base group averages in calculating differences in predicted genetic values because they were an unselected group of animals [9]. Realized yearly genetic gains for milk production and conformation traits were calculated as the regression of average predicted genetic values in the unselected population of cows on time from 1993 through 2012.

Genetic superiority for each trait in each selection path was estimated by averaging the EBVs of sires used or dams used in each birth year of offspring and by taking the differences from the average EBV of the base group born in the same year as sires used or dams used, because where $\text{b}=\frac{\sigma_{\text{G},\text{EBV}}}{\sigma_{\text{EBV}}^{2}}=1$, b is a regression coefficient based on the property of BLUP that the covariance of BLUP EBV with true breeding values is equal to the variance of BLUP EBV [10]. Averages of the EBVs of sires used or dams used are unweighted averages without accounting for differential sire or dam use: that is, all sires and dams are assumed to have the same number of offspring. Generation intervals were computed for each path of selection. The generation interval was defined as the age of the sire or dam of a bull or cow when the offspring was born. Average generation interval was calculated according to progeny birth year. Because the genetic superiority and generation intervals for four paths of selection were measured, the previously intended yearly genetic gain (ΔG/year) for each milk production and conformation trait was computed according to the method of [4]:

(1) $$\Delta \text{G}/\text{year}=\frac{\Delta {\text{G}}_{\text{SB}}+\Delta {\text{G}}_{\text{SC}}+\Delta {\text{G}}_{\text{DB}}+\Delta {\text{G}}_{\text{DC}}}{{\text{L}}_{\text{SB}}+{\text{L}}_{\text{SC}}+{\text{L}}_{\text{DB}}+{\text{L}}_{\text{DC}}}$$

where ΔG_SB_ is average genetic superiority of sires to breed bulls in 1993 through 2012; L_SB_ is average generation interval of sires to breed bulls in 1993 through 2012; and ΔG_SC_, ΔG_DB_, ΔG_DC_, L_SC_, L_DB_, and L_DC_ are defined in the same way as ΔG_SB_ and L_SB_ except for the abbreviations of SC, DB, and DC. The time period of 1993 through 2012 was evaluated as three periods, that is, 1993 through 2003, 2004 through 2012, and 1993 through 2012. The average selection differential, previously intended and actual realized yearly genetic gains were obtained for each of these time periods.

### Index in retrospect

For simplicity, selection was assumed to have been practiced on milk production traits comprising MF and MP yields and on conformation traits comprising FL, TE, and FS. The index of selection (I) is described as:

(2) $$\text{I}=\sum_{\text{i}=1}^{5}{\text{b}}_{\text{i}}{\text{EBV}}_{\text{i}}={\text{b}}_{1}{\text{EBV}}_{\text{MF}}+{\text{b}}_{2}{\text{EBV}}_{\text{MP}}+{\text{b}}_{3}{\text{EBV}}_{\text{FL}}+{\text{b}}_{4}{\text{EBV}}_{\text{TE}}+{\text{b}}_{5}{\text{EBV}}_{\text{FS}}$$

where b_i_ is the coefficient of the index and EBV_i_ is the estimated breeding value for the ith trait in the index. The intended genetic response in the ith trait (ΔG_i_), based on the index of selection, is

(3) $$\Delta {\text{G}}_{\text{i}}=\beta_{\text{G},\text{I}}({\bar{\text{I}}}_{\text{s}}-{\bar{\text{I}}}_{\mu })={\text{G}}_{\text{i}}^{\prime }\text{b}\frac{\left({\bar{\text{I}}}_{\text{s}}-{\bar{\text{I}}}_{\mu }\right)}{\sigma_{\text{I}}^{2}}$$

where β_G_i_I_ is the regression of the genetic value of the ith trait on I; Ī_s_ and Ī_s_ are the mean index values of the population and the selected group, respectively; b is a column vector composed of the coefficients (b_i_, i = 1, …, 5) in (2); G_i_ is the ith column of G corresponding to the ith trait; and G is a (5×5) genetic covariance matrix for the five traits in the index. The components of genetic covariance matrix (G) are estimated from the animal model [8]. The genetic (co)variance components and genetic correlation are shown in Table 1. The difference (Ī_s_ − Ī_μ_)is the selection differential. In contrast, the genetic gain actually realized for each trait is given after the conclusion on selection. The index in retrospect can be obtained from equation (3). That is, because $\frac{\left({\bar{\text{I}}}_{\text{s}}-{\bar{\text{I}}}_{\mu }\right)}{\sigma_{\text{i}}^{2}}$ can be dropped without affecting the proportionality of b,

(4) $${\text{b}}^{*}={\text{G}}^{-1}\Delta$$

where b* is a column vector of actual coefficients in the index in retrospect and Δ is a column vector composed of actually realized yearly genetic gains for the traits of MF, MP, FL, TE, and FS from 1993–2012. Additionally, since the previously intended yearly genetic gain (ΔG/year) from 1993–2012 is calculated from (1) for each trait, index in retrospect to yield these previously intended yearly genetic gains was estimated using the intended genetic gains for the traits of MF, MP,FL, TE, and FS as the components of Δ in (4). In the same way as yearly genetic gain, genetic superiority for the traits of MF, MP, FL, TE, and FS in each path of selection for SB, SC, DB, and DC were used as the components of Δ in (4) to estimate the selection index actually applied for each path of selection. Because the coefficients in the index in retrospect (b*) are coefficients for raw data units, actual coefficients in the index in retrospect are written in standard deviation units (b^s^) to compare weights in index accounting for the different size of genetic variance in each trait. That is, ${\text{b}}_{\text{i}}^{\text{s}}={\text{b}}_{\text{i}}^{*}\sigma_{{\text{G}}_{\text{i}}}$, where σ_G_i__ is the genetic standard deviation for the ith trait, i = MF, MP, FL, TE, and FS. Furthermore, the coefficient in the index in retrospect in standard deviation units (b^s^) is expressed relative to that of milk production yield. That is, relative emphasis on the ith trait compared with milk protein yield (${\text{b}}_{\text{i},\text{MP}}^{\text{s}}$) is written as:

$${\text{b}}_{\text{i},\text{MP}}^{\text{s}}=\frac{{\text{b}}_{\text{i}}^{*}\sigma_{{\text{G}}_{\text{i}}}}{{\text{b}}_{\text{MP}}^{*}\sigma_{{\text{G}}_{\text{MP}}}}.$$

## RESULTS AND DISCUSSION

### Generation intervals and selection differentials

**Figure 1:**
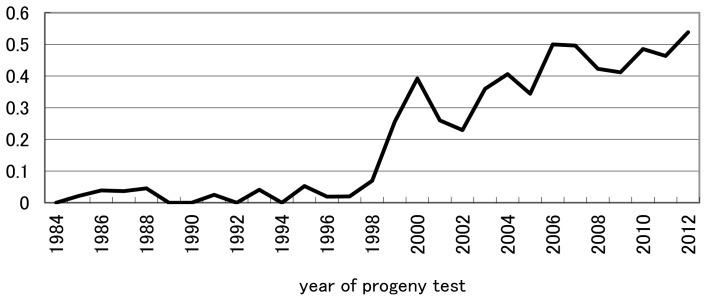
The fraction of dams under 30 month of age to breed young bulls for progeny test.

Generation intervals are shown in Table 2. Generation intervals for dams of cows did not change markedly throughout the study period and were 4.6 yr on average. The generation interval of the path of DC nearly completely agreed with the intervals in Italy [11]. The generation intervals of the paths of SB and SC decreased by approximately 10% from the 1993–2003 period to 2004–2012, and the interval of SB was shorter than that of SC owing to the avoidance of using old proven bulls as sires to breed bulls. The generation interval in the path of DB decreased by approximately 20% from 1993 through 2003 to 2004 through 2012 and was the greatest reduction among the four paths of selection. In the path of DB, National Livestock Breeding Center has produced heifers by multiple ovulation and embryo transfer and has selected them based on records in early stage of their lactations by a random-regression statistical model for estimating the genetic value of a dam’s lifetime milk production [12–15]. As a result, the proportion of DB under 30 month of age increased year by year in Japan (Figure 1) resulting in the reduction in generation interval. Average generation intervals in the four paths of selection decreased from 5.92 yr to 5.42 yr (that is, approximately 10%) from 1993–2003 to 2004–2012.

Standardized genetic superiority or differences in EBV of the selected over the cows in the base group born in the same year as the selected in the four paths of selection, i.e. selection differentials, are shown in Table 3. Selection differentials in milk production traits during 1993–2003 were greater than those of 2004–2012, whereas selection differentials in conformation traits during 1993–2003 were much smaller than those of 2004–2012. These findings indicate that selection was aimed at much greater genetic gain for milk production traits over conformation traits during 1993–2003. In contrast, selection during 2004–2012 had focused on recovering the genetically inferior situation regarding conformation traits during 1993–2003. As a result, the selection differentials had nearly the same magnitude for production and conformation traits during 2004–2012. In comparison, selection regarding milk production and conformation traits along the path of DC was minimal throughout 1993–2012. As a result, replacement rate or fraction of cows from first parity of all the cows in the herd was almost the same (28% to 31%) throughout 1993–2012 in Japan [16].

Regarding average selection differentials throughout the entire study period (1993–2012), differentials in milk production traits were generally greater than those of conformation traits in all four paths of selection; consequently, selection appears to have been aimed to attain greater genetic gain in milk production than in conformation traits during the past two decades in Japan.

### Selection emphasis on milk production and conformation traits in four paths of selection

Coefficients of the index in retrospect relative to those of milk protein yield (${\text{b}}_{\text{i},\text{MP}}^{\text{s}}$) for the four paths of selection (SB, SC, DB, and DC) and three time periods (1993–2003, 2004–2012, and 1993–2012) are shown in Table 4. These coefficients were derived to yield the genetic superiority for milk production (MF and MP) and conformation traits (FL, TE, and FS) in each path of selection (SB, SC, DB, and DC) on the mutual genetic relationship between MF, MP, FL, TE, and FS shown in Table 1. Therefore, negative signs on coefficients arose because of mutual genetic relationship. Emphasis on milk production and conformation traits was computed as the sum of coefficients of MF and MP and as the sum of those of FL, TE, and FS, respectively. Emphasis on conformation traits relative to that on milk production traits is shown as a ratio of the sum of the coefficients of FL, TE, and FS to that of the coefficients of MF and MP. For all time periods from 1993 through 2012, the coefficients of conformation traits were zero in paths of selection for SC and DC. This result indicates that selection of SC and DC focused exclusively on achieving genetic gain for milk production traits. That is, proven sires in the path of SC based on progeny testing were intended to give rise to greater genetic gain in milk production traits in dairy farmers. Questionnaire survey for dairy farmers in 2007 showed that 29% of dairy farmers indicated their intention that milk production traits was the most important traits and 71% of dairy farmers recognized increase in milk production was due to genetic improvement [17]. This tendency of dairy farmers may lead to the above result.

In contrast, the SB and DB paths included a variety of coefficients for milk production and conformation traits, such that selection was intended to yield balanced genetic gains in milk production and conformation traits corresponding to the genetic requirement of each time period. For example, during 1993–2003, when selection emphasized milk production traits, the relative ratio of conformation to milk production traits was 0.08 for the selection path of SB. However, during 2004–2012, when both conformation and milk production traits were emphasized during selection, the relative ratio of conformation to milk production traits was 0.34 for the selection path of SB. For the overall study period of 1993–2012, the relative ratios were the balanced values of 0.39 and 0.51 for the paths of SB and DB, respectively. Additionally, relative emphasis of conformation to milk production traits was slightly greater for the selection path of DB than that of SB throughout the three periods of time. These results indicate that the selection of SB and of DB both focused on balanced selection between milk production and conformation traits during the last two decades in Japan. Selection of SC and of DC turned out to be exclusively based on milk production traits because there are different role allotments for genetic gain between milk production and conformation traits for SB and DB, SC and DC. Consequently each of the four paths of selection has played an individual and important role in Japan. With the development of genomic technology, generation intervals are shortening and thus, young sire arises before the completion of sire’s daughters’ milk production records. Selection of sires and dams to breed sires has played an important role in light of the balanced selection between milk production and conformation traits. Selection of SB and of DB has played an important role throughout the era of progeny testing, i.e., before the genomic era not only in Japan but other countries [9,11]. How to integrate these four paths in the genomic era to increase profitability for dairy farmers and to sustain long-term genetic gain needs to be elucidated.

### Intended and realized yearly genetic gains

**Figure 2:**
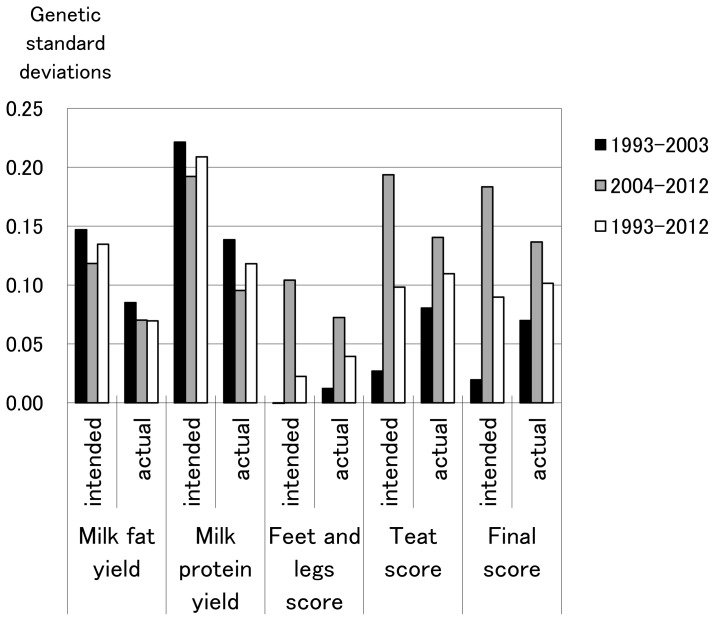
Previously intended and actual yearly genetic gains for milk production and conformation traits (unit = genetic standard deviations).

Yearly genetic gains actually realized and previously intended for milk production and conformation traits in 1993–2003, 2004–2012, and 1993–2012 are shown in Figure 2. Realized and intended yearly genetic gains for milk production traits followed almost the same pattern along with the three time periods of time (1993–2003, 2004–2012, 1993–2012), although realized yearly genetic gain was lower than that intended. Realized genetic gain for conformation traits had almost the same pattern as for intended genetic gain along with the three periods of time as well as milk production traits. On the other hand, overall, for 1993 through 2012, realized annual genetic gain for conformation traits was equivalent to, or greater than, that intended. These findings indicate that realized annual genetic gain in milk production traits was less than intended, whereas realized annual genetic gain for conformation traits was almost the same as, or greater than, the intended gain during the last two decades in Japan. An unfavorable small genetic correlation between milk production and fertility traits was observed [18], and peak milk yield exerted a small and unfavorable genetic effect on fertility traits [19]. Therefore, effects on fertility might lower the realized genetic gain for milk production.

### Selection emphasis on milk production and conformation traits for intended and realized yearly genetic gains

Coefficients of the index in retrospect for previously intended and realized yearly genetic gains for milk production and conformation traits compared with those of MP (${\text{b}}_{\text{i},\text{MP}}^{\text{s}}$) are shown in Table 5. In addition to the coefficients of the index in retrospect, recommended relative weights of milk production and conformation traits in the selection criterion called the Nippon total profit index (NTP) are shown in Table 5. The relative weights for milk production and conformation traits based on indices in retrospect for 1993–2003 in intended yearly genetic gains were 0.87 and 0.13, respectively. Recommended relative weights on milk production and conformation traits in NTP were 0.75 and 0.25, respectively. The intended index in retrospect for 1993–2003 revealed greater weight on milk production traits (0.87) than on conformation traits (0.13) and was about 16% greater than the weight (0.75) of the NTP. However, the actually practiced index in retrospect for 1993–2003 showed that the relative weights of milk production and conformation traits were 0.70 and 0.30, respectively. These results indicate that breeder and dairy farmers used sires and dams in such a way as to emphasize not only milk production but also conformation traits during 1993–2003. Next, during 2004–2012, relative weights of milk production and conformation traits based on indices in retrospect were 0.52:0.48 and 0.46:0.54 for previously intended and actually realized genetic gains, respectively. These results indicate that actual selection was performed nearly as intended during 2004–2012, although relative weight of milk production was slightly lower in actual than in intended. On the other hand, NTP was created in 1995, when relative weights on milk production and conformation traits were 0.75 and 0.25, respectively. Relative weights of milk production and conformation traits in NTP have been 0.7 and 0.3, respectively, throughout 1995 through 2009. In 2010, somatic cell score was included into NTP in which relative weights on milk production, conformation, and somatic cell score were 7.2, 2.4, and 0.4, respectively. Throughout 1995 through 2012 in the years shown in Table 5, recommended relative weights of milk production and conformation traits in NTP have been 0.7 and 0.3, respectively. Therefore, from approximately 2004 through 2012, conformation traits figured more heavily in both intended and actual selection than in the recommended selection criterion of NTP.

**Figure 3:**
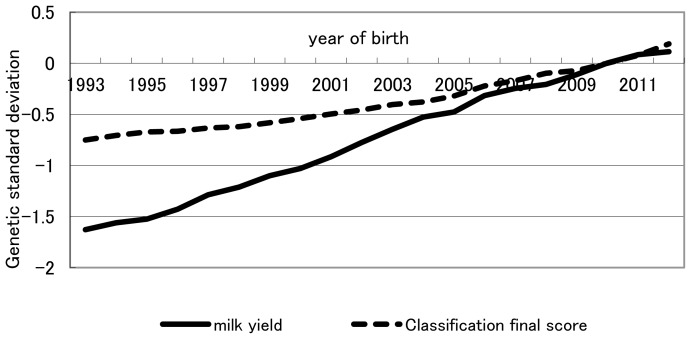
Genetic trend of milk yield and classification final score. Classification final score = (frame×25)+(feet and legs×20)+(dairy strength×15)+(udder×40).

**Figure 4:**
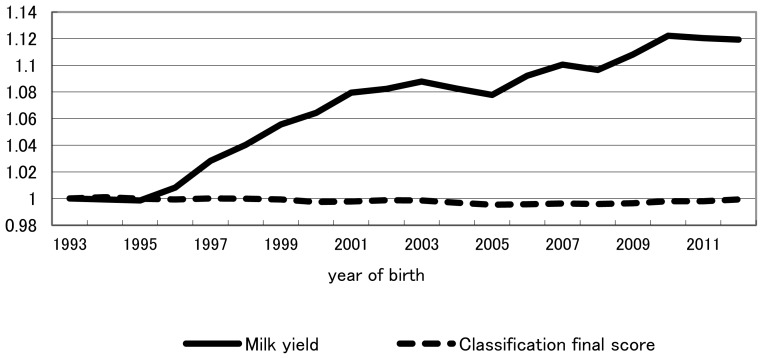
Phenotypic trend of milk yield and classification final score compared with that of 1993. Classification final score = (frame×25)+(feet and legs×20)+(dairy strength×15)+(udder×40).

Finally, throughout the study period as a whole (1993–2012), relative weights of milk production and conformation traits based on indices in retrospect were 0.73:0.27 and 0.56:0.44 for previously intended and actually realized genetic gains, respectively. Overall, throughout the entire study period, realized yearly genetic gain was less than that intended for milk production traits, whereas realized yearly genetic gain was equal to, or greater than, that intended for conformation traits (Figure 1). The trend shown in Table 5 that the increase from 0.27 for the relative weight of conformation traits in the retrospective index in intended genetic gain to 0.44 in actually realized genetic gain likely reflects the trend recognized in Figure 1 for the overall period of 1993–2012. The fact that conformation traits had been more emphasized in actual selection than in intended selection for the past two decades, despite increased recommendation on milk production traits in NTP, indicates that breeder and dairy farmers had a tendency to balance actual selection between milk production and conformation traits. Genetic and phenotypic trends of milk yield and classification final score were plotted in Figures 3, 4, respectively. Genetic trend of milk yield was steeper than that of classification final score (Figure 3). However, regression coefficients of genetic trend of FS on year of birth from 1993 through 2003 and from 2004 through 2012 were 0.0323 and 0.0669, respectively. Regression coefficients of phenotypic trend of milk yield on year of birth from 1993 through 2003 and from 2004 through 2012 were 0.0105 and 0.0057, respectively. Yearly phenotypic increase in milk yield decreased from a period (1993–2003) to a period (2004–2012). On the other hand, phenotypic trend of FS was stable and did not decrease from a period (1993–2003) to a period (2004–2012). This indicates that dairy farmers and breeders put more weight on milk yield than FS on actual selection from 1993 through 2003. On the other hand, during a period from 2004 to 2012, they had a tendency to balance actual selection between milk production and conformation traits. NTP has been created as the desired gain index [20] without calculating economic weight for each trait in the index. It needs investigation whether these tendencies have increased profitability for dairy farmers. In addition, including somatic cell score, reproduction trait, herd life, and lactation persistency among the milk production and conformation traits studied this time and estimating the actually practiced indices in retrospect likely will be informative. Although all information on selection was not included in the index in retrospect studied, including all information would be greatly difficult in a practical sense and the information excluded would have an influence indirectly on selection on milk production and conformation traits. Consequently, relative selection emphasis between milk production and conformation traits obtained retrospectively would be worthwhile as a practical and simple weight.

## CONCLUSION

Selection differentials in milk production traits were generally greater than those of conformation traits in all four paths of selection and throughout the entire study period (1993–2012). These findings imply that selection during the last two decades in Japan emphasized increasing genetic gain in milk production traits over conformation traits. Indices in retrospect for the genetic superiority of sires to breed bulls and cows and of dams to breed bulls and cows revealed that the selection of sires to breed cows and of dams to breed cows exclusively targeted genetic gain for milk production traits, whereas selection of sires to breed bulls and of dams to breed bulls sought balanced selection between milk production and conformation traits. Each of the four paths of selection has played an individual and important role. With shortening generation interval in the genomic era, a young sire arises before the completion of sire’s daughters’ milk production records. How to integrate these four paths of selection in the genomic era is vital. During the study period, the relative weights of milk production and conformation traits based on indices in retrospect were 0.73:0.27 and 0.56:0.44 for previously intended and actually realized yearly genetic gains, respectively. The increase in relative weight for conformation traits from 0.27 in previously intended genetic gain to 0.44 in actually realized genetic gain likely resulted from the tendency of breeder and dairy farmers to balance actual selection between milk production and conformation traits. This study revealed that balanced selection between milk production and conformation traits tends to be favored during actual selection. It merits investigation whether these tendencies have increased profitability for dairy farmers.

## Figures and Tables

**Table 1:** Genetic (co)variance components and correlations between milk fat yield (MF), milk protein yield (MP), feet and legs score (FL), teat score (TE), and classification final score (FS)1)2)3)

Items	MF	MP	FL	TE	FS4)
MF	857	366.39	−2.451	1.54	−3.196
MP	0.625	401	−0.591	2.394	−0.718
FL	−0.122	−0.043	0.471	0.404	0.378
TE	0.066	0.15	0.739	0.635	0.528
FS	−0.14	−0.046	0.707	0.849	0.608

1) Diagonal and upper triangle: genetic (co)variance components.

2) Unit of milk production traits is kg and that of conformation traits is score.

3) Lower triangle: genetic correlation.

4) FS (final score) = (frame×25)+(feet and legs×20)+(dairy strength×15)+(udder×50).

**Table 2:** Generation intervals (years) as average ages of parents when offspring were born

Items	1993–2003	2004–2012	1993–2012
SB	6.85	6.22	6.56
SC	7.87	7.34	7.64
DB	4.33	3.59	4.00
DC	4.62	4.53	4.58
Average	5.92	5.42	5.69

SB, path from sire to bull; SC, path from sire to cow; DB, path from dam to bull; DC, path from dam to cow.

**Table 3:** Standardized genetic superiority or differences in estimated breeding values of the selected over cows in the base group born in the same year as the selected in the four paths of selection1)

Items	Milk fat yield	Milk protein yield	Feet and legs score	Teat score	Classification final score2)
1993–2003					
SB	1.368	2.136	−0.066	0.356	0.275
SC	1.152	1.689	−0.829	−0.040	−0.088
DB	0.862	1.315	0.032	0.579	0.486
DC	0.094	0.100	−0.059	−0.259	−0.210
2004–2012					
SB	0.993	1.624	0.866	1.758	1.609
SC	0.726	1.299	0.767	1.261	1.163
DB	0.792	1.174	0.587	1.123	1.146
DC	0.052	0.069	0.039	0.056	0.057
1993–2012					
SB	1.200	1.906	0.354	0.987	0.875
SC	0.960	1.514	−0.111	0.546	0.475
DB	0.831	1.252	0.282	0.824	0.783
DC	0.075	0.086	−0.015	−0.118	−0.090

SB, path from sire to bull; SC, path from sire to cow; DB, path from dam to bull; DC, path from dam to cow.

1) Data are shown as genetic standard deviations.

2) Classification final score = (frame×25)+(feet and legs×20)+(dairy strength×15)+(udder×40).

**Table 4:** Coefficients of the index in retrospect relative to those of milk protein yield for four paths of selection (SB, SC, DB, and DC) and three time periods (1993–2003, 2004–2012, and 1993–2012)

Items	MF	MP	MF+MP	FL	TE	FS1)	FL+TE+FS	Conformation/ milk production
1993–2003								
SB	0.115	1	1.115	−0.062	−0.526	0.677	0.089	0.080
SC	2.329	1	3.329	0.000	0.000	0.000	0.001	0.000
DB	0.130	1	1.130	−0.454	−0.060	0.825	0.311	0.275
DC	2.494	1	3.494	0.000	0.000	0.000	0.001	0.000
2004–2012								
SB	0.129	1	1.129	−0.461	0.427	1.129	1.094	0.343
SC	2.226	1	3.226	0.000	0.000	0.000	0.001	0.000
DB	0.320	1	1.320	−0.321	−0.225	1.588	1.042	0.790
DC	2.384	1	3.384	0.000	0.000	0.000	0.001	0.000
1993–2012								
SB	0.120	1	1.120	−0.202	−0.192	0.835	0.441	0.394
SC	2.291	1	3.291	0.000	0.000	0.000	0.001	0.000
DB	0.208	1	1.208	−0.399	−0.127	1.138	0.611	0.506
DC	2.457	1	3.457	0.000	0.000	0.000	0.001	0.000

SB, path from sire to bull; SC, path from sire to cow; DB, path from dam to bull; DC, path from dam to cow; MF, milk fat yield; MP, milk protein yield; FL, feet and legs score; TE, teat score; FS, classification final score.

1) FS (final score) = (frame×25)+(feet and legs×20)+(dairy strength×15)+(udder×40).

**Table 5:** Coefficients of the index in retrospect for previously intended and actual yearly genetic gains for milk production and conformation traits relative to those of milk protein yield1)

Items	MF	MP	MF+MP	FL	TE	FS2)	FL+TE+FS	Milk production/ total coefficients	Confirmation/ total coefficients
Intended									
1993–2003	0.382	1	1.382	−0.472	−2.037	2.720	0.211	0.868	0.132
2004–2014	0.132	1	1.132	−0.269	0.322	0.969	1.023	0.525	0.475
1993–2014	0.313	1	1.313	−0.413	−1.348	2.248	0.487	0.730	0.270
Actual									
1993–2003	0.059	1	1.059	−0.449	0.118	0.794	0.463	0.696	0.304
2004–2014	0.553	1	1.553	−0.641	0.536	1.949	1.844	0.457	0.543
1993–2014	0.057	1	1.057	−0.520	0.322	1.027	0.828	0.561	0.439

MF, milk fat yield; MP, milk protein yield; FL, feet and legs score; TE, teat score; FS, classification final score.

1) Recommended relative weights of milk production and conformation traits in the selection criterion called Nippon Total Profit Index (NTP) have been 0.75 and 0.25, respectively, throughout 1993 through 2014.

2) FS (final score) = (frame×25)+(feet and legs×20)+(dairy strength×15)+(udder×40).
